# Interrelation between job satisfaction and self-regulation resources in professionals under work stress due to the COVID-19 pandemic

**DOI:** 10.1192/j.eurpsy.2021.822

**Published:** 2021-08-13

**Authors:** M. Titova

**Affiliations:** Department Of Psychology, Lomonosov Moscow State University, Russian Federation

**Keywords:** COVID-19, stress, anxiety, job satisfaction, self-regulation resources, coping behaviour

## Abstract

**Introduction:**

The problem of job satisfaction connected with work efficiency and psychological well-being of professionals is especially actual in stress conditions due to COVID-19 pandemic. The stress has a negative effect on job satisfaction (Singh et al., 2019). The one of the most important criteria for successful activities is a job satisfaction (Burić & Moè, 2020). The highest level of job satisfaction can be achieved by the high adaptive potential of professionals, who have a diverse arsenal of self-regulation resources and apply them adequately to working conditions (Kuznetsova et al., 2019).

**Objectives:**

The study was held in 45 professionals from different fields, who work remotely during the self-isolation due to COVID-19 pandemic and aimed to estimate the correlation between job satisfaction and self-regulation resources of professionals under work stress.

**Methods:**

The assessment methods included: 1) test “Job Satisfaction” by V.A. Rozanova 2) S. Hobfoll’s “SACS” 3) Ch. Spilberger’s “Trait Anxiety”.

**Results:**

The results revealed an average level of anxiety with a tendency to growth. Avoidance, asocial and aggressive behaviour are frequently used. The direct correlation between the level of job satisfaction and such a resource of self-regulation as a search for social contact was revealed (r=0.291; p=0.049). The general level of anxiety is directly related to avoidance (r=0. 374; p=0.011), manipulative (r=0.343; p=0.021) and aggressive actions (r=0.343; p=0.021), and negatively correlates with assertive actions (r=-0.703; p=0).

**Conclusions:**

The results of the study can be used to develop programs to improve the psychological well-being and performance of employees working under stress due to COVID-19 pandemic.

